# Alterations of the vaginal microbiome in healthy pregnant women positive for group B Streptococcus colonization during the third trimester

**DOI:** 10.1186/s12866-022-02730-8

**Published:** 2022-12-21

**Authors:** Sarah Shabayek, Asmaa M. Abdellah, Mohammed Salah, Mohammed Ramadan, Nora Fahmy

**Affiliations:** 1 Department of Microbiology and Immunology, Faculty of Pharmacy, Suez Canal University, Ismailia, Egypt; 2 Department of Obstetrics and Gynecology, Faculty of Medicine, Suez Canal University, Ismailia, Egypt; 3 Department of Microbiology and Immunology, Faculty of Pharmacy, Port Said University, Port Said, Egypt; 4 Department of Microbiology and Immunology, Faculty of Pharmacy, Al-Azhar University, Assiut, Egypt

**Keywords:** Vagina, Microbiome, Group B Streptococcus, *Streptococcus agalactiae*, Pregnant, Third trimester, Healthy

## Abstract

**Background:**

*Streptococcus agalactiae* or group B Streptococcus (GBS) asymptomatically colonizes the genitourinary tracts of up to 30% of pregnant women. Globally, GBS is an important cause of neonatal morbidity and mortality. GBS has recently been linked to adverse pregnancy outcomes. The potential interactions between GBS and the vaginal microbiome composition remain poorly understood. In addition, little is known about the vaginal microbiota of pregnant Egyptian women.

**Results:**

Using V3-V4 16S rRNA next-generation sequencing, we examined the vaginal microbiome in GBS culture-positive pregnant women (22) and GBS culture-negative pregnant women (22) during the third trimester in Ismailia, Egypt. According to the alpha-diversity indices, the vaginal microbiome of pregnant GBS culture-positive women was significantly more diverse and less homogenous. The composition of the vaginal microbiome differed significantly based on beta-diversity between GBS culture-positive and culture-negative women. The phylum *Firmicutes* and the family *Lactobacillaceae* were significantly more abundant in GBS-negative colonizers. In contrast, the phyla *Actinobacteria*, *Tenericutes*, and *Proteobacteria* and the families *Bifidobacteriaceae*, *Mycoplasmataceae*, *Streptococcaceae*, *Corynebacteriaceae*, *Staphylococcaceae*, and *Peptostreptococcaceae* were significantly more abundant in GBS culture-positive colonizers. On the genus and species levels, *Lactobacillus* was the only genus detected with significantly higher relative abundance in GBS culture-negative status (88%), and *L. iners* was the significantly most abundant species. Conversely, GBS-positive carriers exhibited a significant decrease in *Lactobacillus* abundance (56%). In GBS-positive colonizers, the relative abundance of the genera *Ureaplasma*, *Gardnerella*, *Streptococcus*, *Corynebacterium*, *Staphylococcus*, and *Peptostreptococcus* and the species *Peptostreptococcus anaerobius* was significantly higher. The Kyoto Encyclopedia of Genes and Genomes (KEGG) pathways related to the metabolism of cofactors and vitamins, phosphatidylinositol signaling system, peroxisome, host immune system pathways, and host endocrine system were exclusively enriched among GBS culture-positive microbial communities. However, lipid metabolism KEGG pathways, nucleotide metabolism, xenobiotics biodegradation and metabolism, genetic information processing pathways associated with translation, replication, and repair, and human diseases (*Staphylococcus aureus* infection) were exclusively enriched in GBS culture-negative communities.

**Conclusions:**

Understanding how perturbations of the vaginal microbiome contribute to pregnancy complications may result in the development of alternative, targeted prevention strategies to prevent maternal GBS colonization. We hypothesized associations between inferred microbial function and GBS status that would need to be confirmed in larger cohorts.

**Supplementary Information:**

The online version contains supplementary material available at 10.1186/s12866-022-02730-8.

## Background

The human microbiome at various body sites defines the host’s metabolic features and plays an essential role in maintaining the homeostasis of the host immune system and resisting invading pathogens [1–3]. Thus, the impact of lifestyle and environmental factors on commensal microbial communities in healthy and diseased individuals could be better understood by examining microbiota variations. These findings are extended in an effort to elucidate the complex interplays between the host and the associated microbiome [1–3].

The vaginal mucosal surface is one of the primary entry points for invading pathogens and, consequently, a principal immunological barrier [4]. *Streptococcus agalactiae*, or group B Streptococcus (GBS), is an encapsulated Gram-positive pathobiont that colonizes the vaginal mucosa and/or rectum of up to 30% of healthy women asymptomatically [5]. GBS is an important cause of morbidity and mortality in neonates worldwide [5–8]. Through ascending uterine infection or during labor, the maternal carriage is a predisposing factor for potential neonatal disease [5]. In addition, an increasing proportion of GBS infections are reported in pregnant women who are colonized [8–11]. Recently, GBS has been linked to adverse pregnancy outcomes, including stillbirths and preterm births [6, 8, 12, 13]. To prevent perinatal GBS disease, the current guidelines issued by the Centers for Disease Control and Prevention (CDC) recommend culture-based maternal screening during the third trimester and intrapartum antibiotic prophylaxis [5].

*Lactobacillus* dominance is a key feature of a healthy vaginal microbiome. They are essential for restoring the vaginal ecosystem and maintaining a low pH, which functions as a barrier against invading and colonizing pathogens [4]. The vagina is susceptible to bacterial vaginosis, genital tract infections, urinary tract infections and vulvovaginal candidiasis if the *Lactobacillus* population is diminished or eradicated [4, 14]. During pregnancy, there has been a marked reduction in species diversity of the vaginal microbiome, where the dominance of a single *Lactobacillus* species has been widely reported [15]. This is thought to be essential for protecting both mother and fetus against infection [15]. Five distinct community state types (CSTs) have been established for the vaginal microbiome of healthy women [16]. Four of these CSTs are dominated by one *lactobacillus* species; *L. crispatus* dominated CST-I, *L. gasseri* dominated CST-II, *L. iners dominated* CST-III, and *L. jensenii* dominated CST-V. However, the scarcity of lactobacilli with numerous strict and facultative anaerobes was a distinctive feature of the *Lactobacillus*-poor CST-IV [16].

Previous reports have investigated the impact of GBS colonization on the vaginal microbiota using classical culture-based methods [17–21]. These microbiological techniques are restricted to culturable microbes, which account for only 10% of the human microbiome. However, with the use of high-throughput next-generation sequencing technologies, culture-independent, large-scale investigations of the vaginal microbiome in relation to GBS carriage can be conducted with unprecedented taxonomic resolution [22–26]. This allows for the detection of low-abundance, non-culturable microbes.

GBS is an opportunistic vaginal pathobiont whose life cycle involves a transformation from a harmless commensal state to an invading pathogen for both mother and infant [27–29]. Thus, it could be hypothesized that the response of vaginal communities to dysbiosis is linked to differences in species composition. Variations in vaginal microbiota have been linked to complications during pregnancy [15]. The potential interactions between GBS and the composition of vaginal bacterial communities are poorly understood. Furthermore, there is a lack of literature on the subject. Additionally, the microbial composition of vaginal communities varies significantly among women. It is viewed as a composite that is influenced by several factors, including demographics, sampling sites, sequencing technologies, etc. [15]. Ethnicity was found to be a significant factor in determining the structure of the vaginal microbiome [28, 30]. Consequently, it may also be geographically distinct. Except for a recent study on bacterial vaginosis [31], no other studies on the vaginal microbiome in Egypt are available.

Using 16S rRNA next-generation sequencing technology and advanced microbiome profiling, we compared the vaginal microbial communities of GBS culture-positive pregnant women with those of GBS culture-negative pregnant women during the third trimester in Ismailia, Egypt. We were able to gain a greater understanding of taxonomy and microbial diversity and predict the functional pathways associated with GBS carriage.

## Results

### Demographic data and 16S sequences characteristics

Vaginal swabs collected from 44 pregnant subjects were sequenced by Illumina MiSeq platform and used for 16S rRNA microbiome analysis. These included 22 GBS culture-positive women and 22 GBS culture-negative women. The ages of the participants ranged from 19 to 44 years, with an average age of 31.11 ± 7.41 years, 31.18 ± 6.62 for GBS culture-positive pregnant women, and 31.05 ± 8.30 for GBS culture-negative pregnant women. Furthermore, the mean parity was 2.90 ± 1.41; 3.18 ± 1.26 for GBS culture-positive pregnant women and 2.64 ± 1.53 for GBS culture-negative pregnant women. There was no significant difference in age and parity of participants between the two groups Table 1. Sequence processing and taxonomic assignment resulted in 3,299,595 reads (average counts per sample 74, 990) and 1793 OTUs as revealed by Greengenes versus 2215 OTUs by SILVA.

**Table 1:** Characteristic of participants recruited for 16S rRNA microbiome analysis

GBS status (number)	Age (mean ± SD)	***p value***	Parity	***p value***
Culture-positive (22)	31.18 ± 6.62	0.952 0.952 (Welch’ t test)	3.18 ± 1.26	0.203 0.203 (Welch’s t test)
Culture-negative (22)	31.05 ± 8.30		2.64 ± 1.53	

Two-tailed unpaired t-test and Welch’s two-sample t-test were calculated to evaluate the statistical differences between GBS culture-positive and GBS culture-negative participants for age and parity. *P* value < 0.05 is considered statistically significant

### Alpha-diversity

**Fig. 1:**
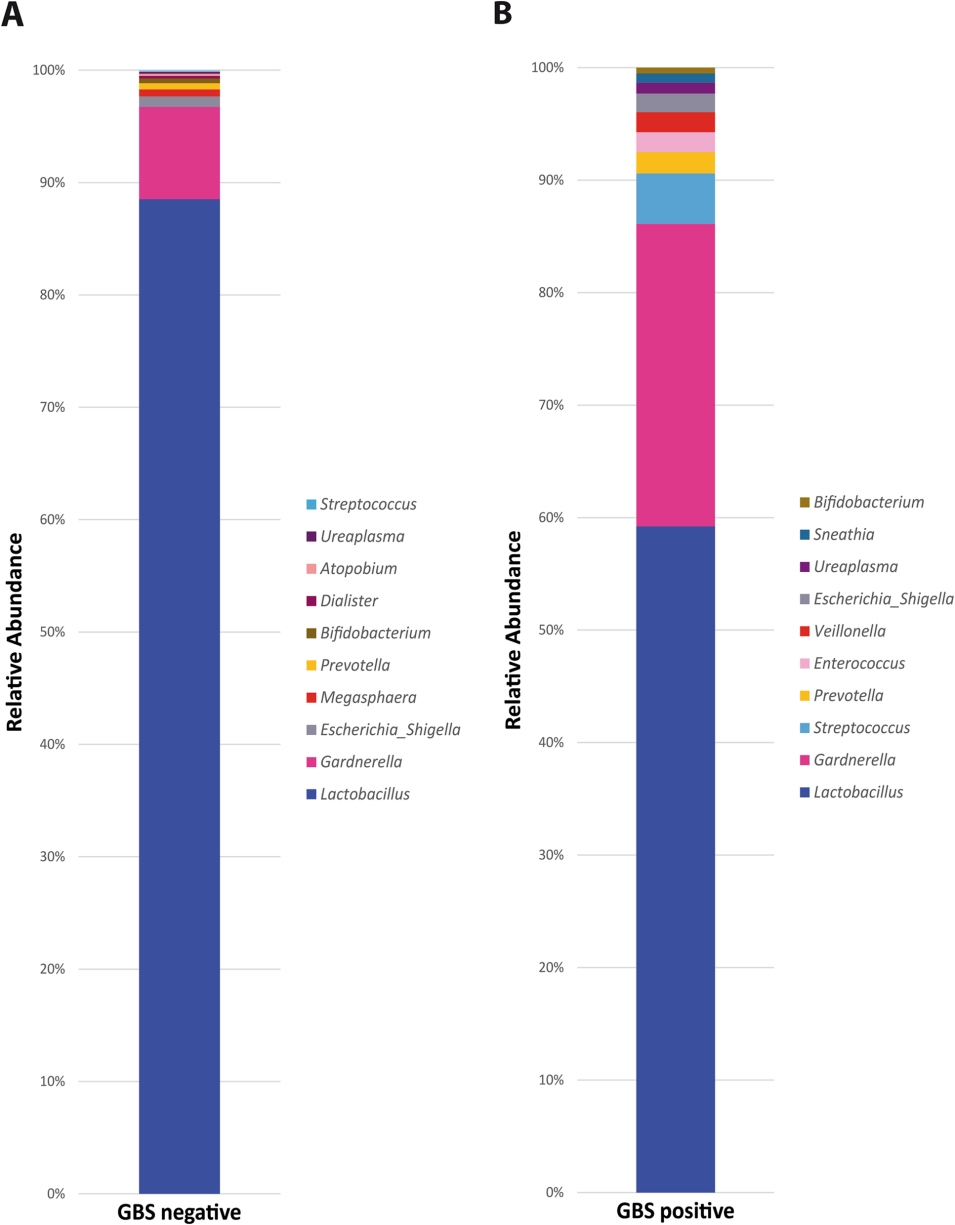
Relative abundance of the 10 top vaginal bacterial community genera in pregnant Egyptian women during the third trimester according to GBS status using SILVA database. **A** Relative abundance of vaginal genera in GBS culture-negative pregnant women. **B** Relative abundance of vaginal genera in GBS culture-positive pregnant carriers

Alpha-rarefaction indicated sufficient sequencing depth (Supplementary Fig. 1). The vaginal microbial communities of GBS culture-positive pregnant women tended to be significantly more diverse and less homogeneous than those of GBS culture-negative pregnant women, as defined by Shannon (*p* values = 0.0033461) and Simpson (*p* values = 0.0004057) alpha-diversity indices but not by chao1 (*p* value = 0.086554). As expected, *Lactobacillus* was nearly the only genus among GBS culture-negative pregnant women (88%), whereas GBS culture-positive pregnant women had a less homogeneous microbial structure with a significant decrease in *Lactobacillus* abundance (56%) (Fig. 1). Alpha diversity is shown in Supplementary Fig. 2.

### Beta-diversity

**Fig. 2:**
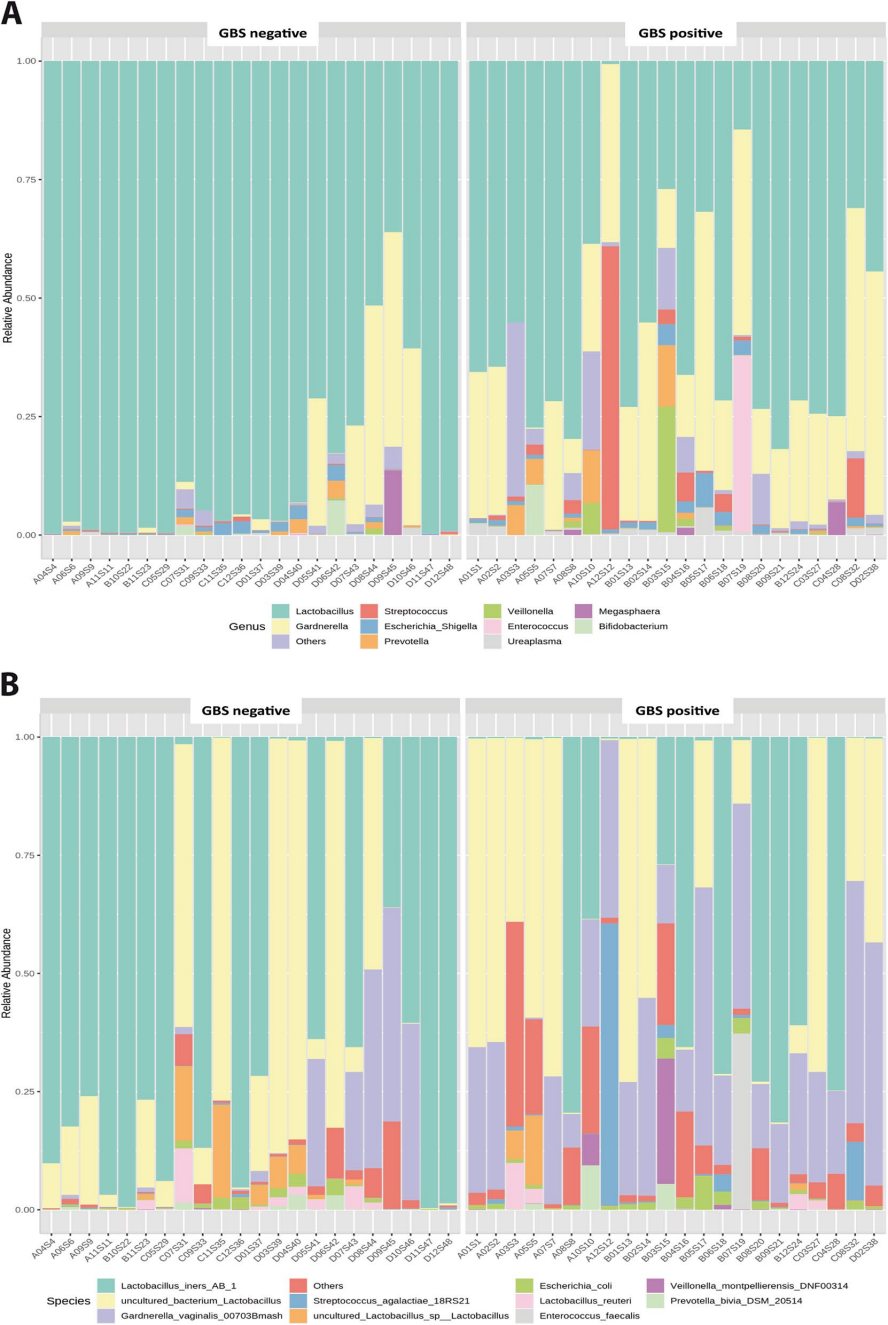
Taxa level relative abundance in vagina of pregnant Egyptian women during the third trimester according to GBS status using SILVA database. **A** Stacked bar charts represent relative proportions of the 10 most predominant genera in the vaginal microbiome of GBS culture-negative pregnant women (left) and GBS culture-positive pregnant carriers (right). **B** Stacked bar charts represent relative proportions of the 10 most predominant species in the vaginal microbiome of GBS culture-negative pregnant women (left) and GBS culture-positive pregnant carriers (right). Each bar represents one sample

We inferred differences in microbiota profiles using Bray–Curtis and Jaccard dissimilarity indices. We observed a significant difference in the composition of vaginal microbiome between GBS culture-positive and GBS culture-negative pregnant women as assessed by PERMANOVA using the Bray–Curtis and Jaccard dissimilarity indices (*p* value < 0.001). Beta diversity is shown in Supplementary Figs. 3. Vaginal community composition on genus and species level is shown in Figs. 2 as revealed by SILVA database.

### The core microbiome, CST classification, and taxa biomarker analysis based on GBS status

In general, similar taxonomic assignments were revealed by Greengenes, SILVA, and Greengenes-based Illumina 16S rRNA microbiome analysis software. Species taxa level assignment power was variable where best taxonomic assignations were provided by SILVA and Illumina 16S rRNA microbiome analysis software. However, no obvious discrepancies were detected, and all data could be combined and integrated to provide better species assignments of vaginal bacterial communities.

Regardless of GBS status, the core vaginal microbiome was dominated by the phyla *Firmicutes* and, to a lesser extent, *Actinobacteria*. The most abundant families were *Lactobacillaceae* and *Bifidobacteriaceae*. The most predominant genera were *Lactobacillus*, followed by *Gardnerella*. The species with the highest abundance was *L. iners.*

Samples could be classified into 7 CST groups. Two samples were not designated (No types). These belonged to the GBS-positive vaginal communities. *L. iners* dominant CST-III was the dominant community state representing 52.2% (23/44), this was followed by *L. crispatus* dominant CST-I (6/44, 13.6%), *L. johnsonii* dominant CST-Other (5/44, 11.3%), CST-VII (4/44, 9%), *L. jensenii* dominant CST-V (2/44, 4.5%), and finally *L. gasseri* dominant CST-II (1/44, 2.2%) and *Lactobacillus*-poor CST-IV (1/44, 2.2%). CST-III and CST-I represented more the 85% (19/22) of GBS-negative communities. The remaining 15% (3/22) was represented as CSTs II, V and VII (One sample for each CST). In contrast, CST-III and CST-I represented less than half of the GBS-positive communities (10/22, 45.5%). *L. johnsonii* dominant CST-Other was exclusive for GBS-positive communities representing more the 20% (5/22, 22.7%). The remaining samples were classified as CST-VII (3/22, 13.6%), CST-I (2/22, 9%), CST-V (1/22, 4.5%), and CST-IV (1/22, 4.5%). None of the samples was assigned to CST-VI. Distribution of samples by GBS status across CSTs is shown in Table 2. Species taxa level relative abundance of dominant *Lactobacillus* species in vagina of pregnant Egyptian women during the third trimester according to GBS status as revealed by Illumina 16S rRNA microbiome individual reports is shown in Supplementary Fig. 4.

**Table 2:** Distribution of samples by GBS status across community states (CST)

CST	GBS-positive	GBS-negative	Total
I	2	4	6
II	–	1	1
III	8	15	23
IV	1	–	1
V	1	1	2
VI	–	–	–
VII	3	1	4
Other (*L. johnsonii* dominant)	5	–	5
No type	2	–	2
Total	22	22	44

**Fig. 3:**
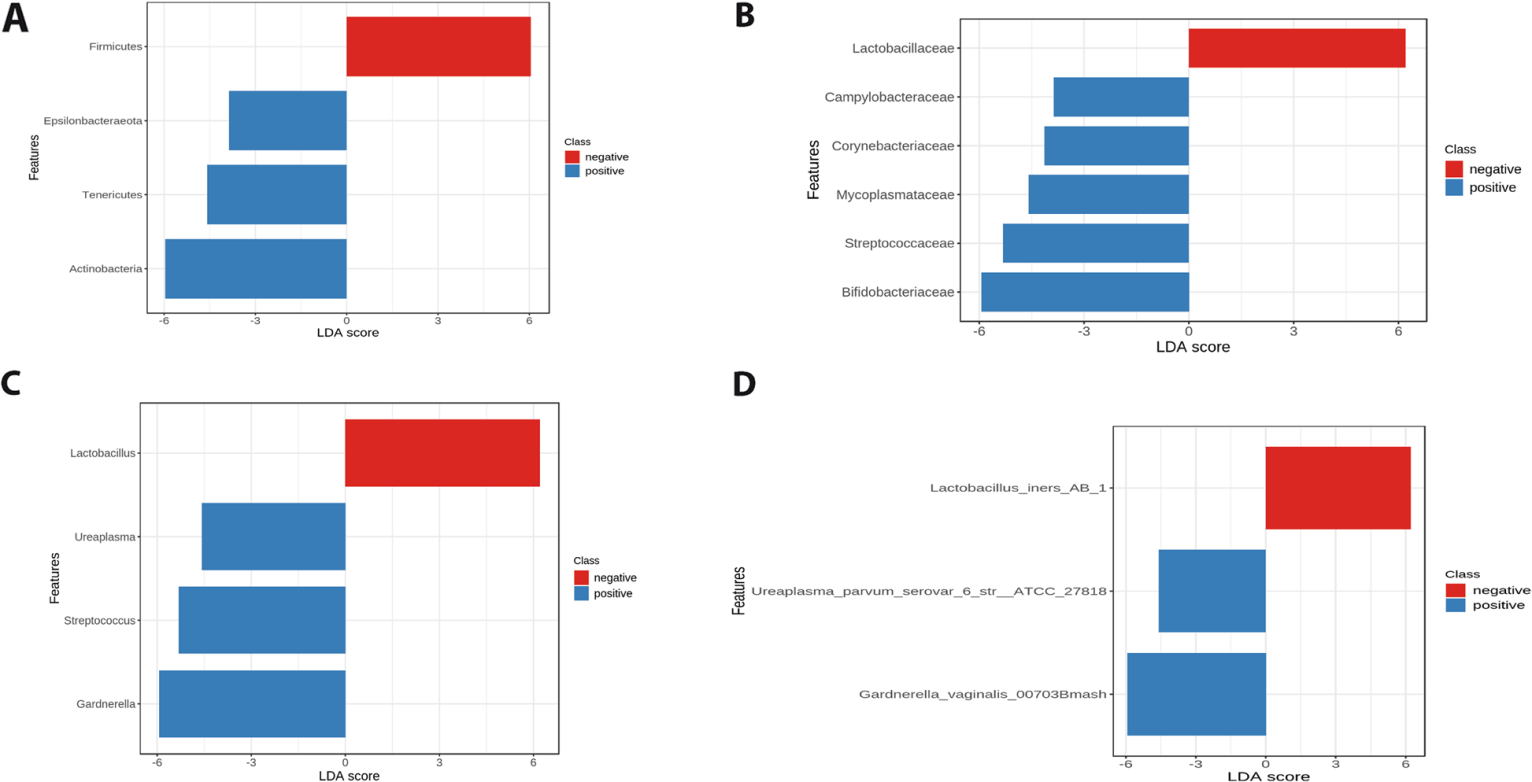
Linear discriminant analysis (LDA) effect size (LEfSe) biomarker analysis showing biomarker phylum (**A**), family (**B**), genus (**C**), and species (**D**) with significant differential abundance in GBS culture-negative and GBS culture-positive pregnant Egyptian women using SILVA database. FDR adjusted *p* values and LDA score > 2 was considered statistically significant. Not_Assigned refers to taxa that were challenging for biological interpretation

**Fig. 4:**
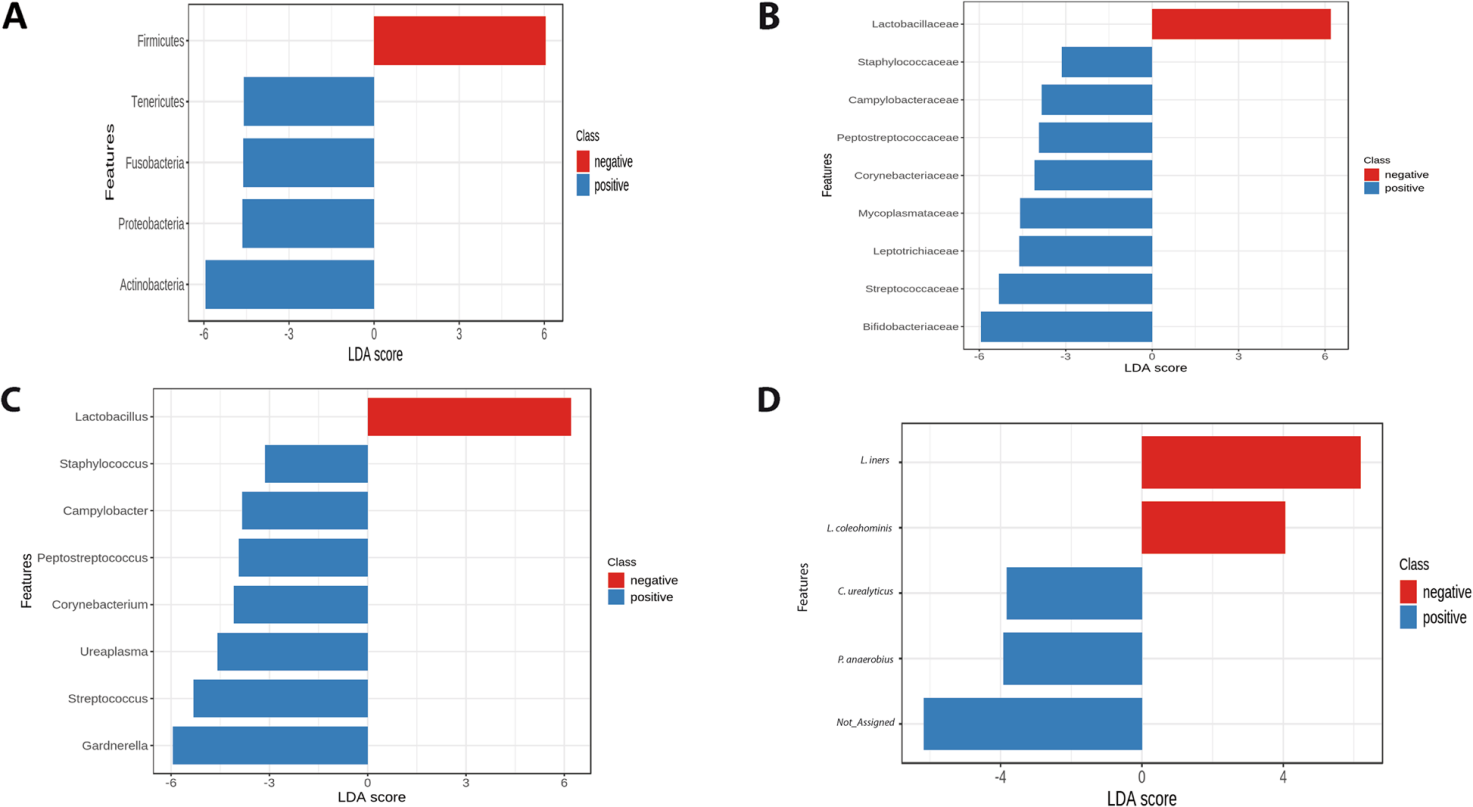
Linear discriminant analysis (LDA) effect size (LEfSe) biomarker analysis showing biomarker phylum (**A**), family (**B**), genus (**C**), and species (**D**) with significant differential abundance in GBS culture-negative and GBS culture-positive pregnant Egyptian women using Greengenes database. FDR adjusted *p* values and LDA score > 2 was statistically significant. Not_Assigned refers to taxa that were challenging for biological interpretation

We used MicrobiomeAnalyst’s LEfSe analysis tool to identify potential taxa biomarkers based on GBS status. LEfSe was used to identify taxa that were significantly different between GBS culture-positive and GBS culture-negative colonizers (Figs. 3 and 4 panels A, B, C, and D). The genera *Ureaplasma*, *Gardnerella*, *Streptococcus*, *Corynebacterium*, *Staphylococcus*, *Peptostreptococcus,* and *Campylobacter* (Fig. 3C, Fig. 4C) and the species *Gardnerella vaginalis*, *Peptostreptococcus anaerobius, Campylobacter ureolyticus, Ureaplasma parvun serovar 6* (Fig. 3D, Fig. 4D) exhibited a significantly greater relative abundance in GBS culture-positive colonizers, whereas *Lactobacillus* was the only genus detected by LEfSe with significantly higher relative abundance in GBS culture-negative status (Fig. 3C, Fig. 4C), and *L. iners and Lactobacillus coleohominis* were the significantly more abundant species (Fig. 4D).

### Functional alterations in the vaginal flora

**Fig. 5:**
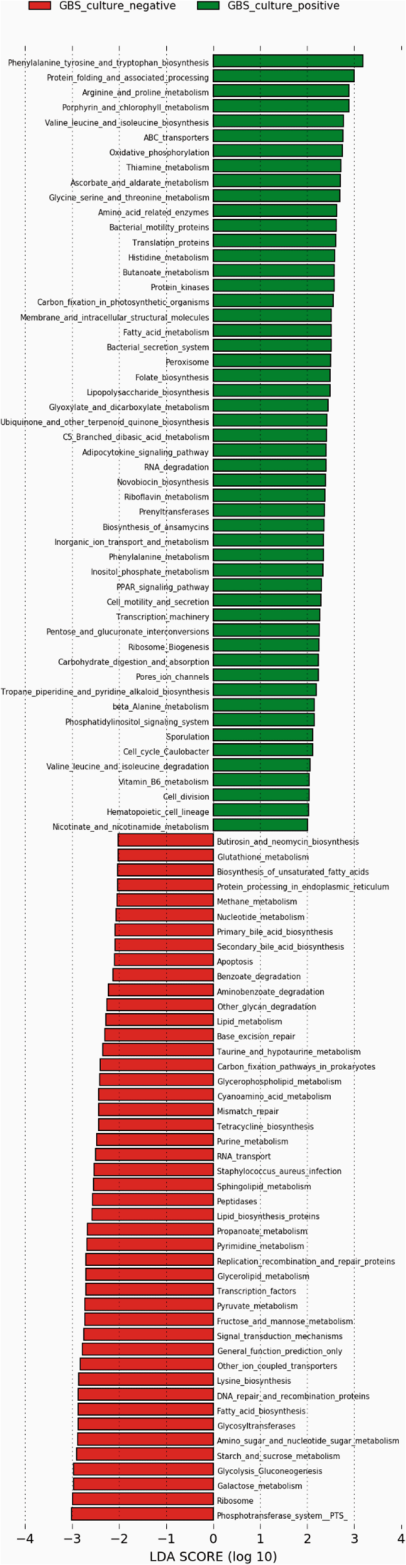
Linear discriminant analysis (LDA) effect size (LEfSe) analysis of differentially abundant pathways between GBS-positive (green) and GBS-negative (red) bacterial communities during the third trimester of pregnancy in Egypt. LDA scores > 2 were considered statistically significant

In order to analyze the functional capacities of the vaginal microbiota among GBS culture-positive and culture-negative pregnant individuals, we predicted KOs (Kyoto Encyclopedia of Genes and Genomes (KEGG) Orthology groups) from the 16S rRNA gene data. Predictive functional profiling of microbial communities revealed that 52 KEGG pathways were significantly enriched in GBS culture-positive microbial communities compared to 46 KEGG pathways in GBS culture-negative microbial communities (Fig. 5, Table 3).

**Table 3:** Differentially abundant KEGG pathways between GBS-positive and GBS-negative bacterial communities during the third trimester of pregnancy in Egypt

GBS-Positive	GBS-Negative
**Exclusively enriched pathways**	
**Metabolism of cofactors and vitamins** (Thiamine metabolism, Riboflavin metabolism, Vitamin B6 metabolism, nicotinate and nicotinamide metabolism, folate biosynthesis, porphyrin metabolism, ubiquinone and other terpenoid biosynthesis).	**Lipid metabolism** (biosynthesis of fatty acid, primary bile acid, secondary bile acid, and unsaturated fatty acids, metabolism of glycerolipids, glycerophospholipids, and sphingolipids).
**Signal transduction** (phosphatidylinositol signaling system).	**Nucleotide metabolism** (purine and pyrimidine metabolism).
**Peroxisome**.	**Xenobiotics biodegradation and metabolism** (benzoate and aminobenzoate degradation).
**Host immune system pathways** (hematopoietic cell lineage).	**Genetic information processing pathways** (translation, replication, and repair).
**Host endocrine system** (adipocytokine signaling pathway, PPAR signaling pathway).	**Human diseases** (*Staphylococcus aureus* infection).
**Enriched Pathways related to:**	
**Amino acid metabolism**	
Glycine, serine, and threonine metabolism, arginine and proline metabolism, histidine metabolism, phenylalanine metabolism, beta-alanine metabolism, phenylalanine, tyrosine, and tryptophan biosynthesis, and valine, leucine, and isoleucine biosynthesis	Lysine biosynthesis, taurine, and hypotaurine metabolism, cynoamino acid metabolism, glutathione metabolism
**Carbohydrate metabolism**	
Ascorbate and aldarate metabolism, glyoxylate, and dicarboxylate metabolism, butanoate metabolism, C5-branched dibasic acid metabolism, inositol phosphate metabolism, pentose, and glucuronate interconversions	Metabolism of fructose and mannose, galactose, starch and sucrose, amino sugar and nucleotide sugar, pyruvate, propanoate, and glycolysis/gluconeogenesis
**Glycan biosynthesis and degradation**	
Lipopolysaccharide biosynthesis	Glycan degradation
**Metabolism of trepenoids and polyketides**	
Biosynthesis of ansamycins, novobiocin, tropane, piperidine, and pyridine alkaloid.	Tetracycline biosynthesis.
**Membrane transport**	
ABC transporters and bacterial secretion systems	Phosphotransferase system (PTS)
**Energy metabolism**	
Oxidative phosphorylation and carbon fixation in photosynthetic organisms.	Methane metabolism and carbon fixation in prokaryotes.

## Discussion

Maternal colonization is a significant risk factor for neonatal GBS transmission and potentially adverse pregnancy outcomes [5, 6]. Although GBS colonization is asymptomatic and harmless in healthy women, serious infections were reported in pregnant mothers as well [6, 8–11]. In most instances, GBS is a harmless commensal of the vaginal microbiota and does not cause invasive disease [28]. However, the circumstances that favor GBS reversion from the commensal to the invasive state remains misunderstood. For neonatal sepsis and meningitis, environmental pH appears to surrogate GBS switch from harmless to invasive as the bacterium migrates from the acidic vagina to the neutral blood [27, 29, 32, 33]. Successful GBS colonization of the vagina is inversely related to vaginal *Lactobacillus* populations which consequently results in creating a dysbiotic environment with increased vaginal pH [28, 34]. Such environment would be permissive to the overgrowth of urogenital pathogens including GBS. Hence, as a transient vaginal pathobiont, GBS can revert from asymptomatic carriage under acidic conditions to invasive state and resistance to host immunity under neutral conditions. Environmental pH fluctuations from acidic to neutral pH up-regulates the expression of several virulence genes facilitating GBS switch to virulent state. One of the most important virulence factors is the beta-hemolysin/cytolysin which is up-regulated upon GBS transition from acidic to neutral conditions through the two-component signal transduction system CovRS [32]. Beta-hemolysin/cytolysin is crucial for GBS infection and immune evasion. It is essential in promoting GBS dissemination in uterine, placental and fetal tissues during pregnancy [35].

As previously reported [4, 15, 30, 36, 37], our results identified *Lactobacilli* as the sole member of the vaginal community among pregnant women during the third trimester. Pregnant women have a significantly higher prevalence of *Lactobacillus* vagitypes and a proportionally lower prevalence of vagitypes dominated by other taxa, according to [30]. This is consistent with the notion that pregnancy promotes the dominance of *Lactobacillus* at the expense of other taxa, resulting in a vaginal microbiome ecosystem that is less complex [30]. *L. iners* was the most predominant *Lactobacillus* species in the vaginal microbiome of our pregnant subjects regardless of GBS status. According to [16], this was classified as a CST-III vaginal community state. This observation was supported by [38]. Throughout the three trimesters of pregnancy, a longitudinal study was conducted to characterize the cervicovaginal microbiome of pregnant women. They demonstrated that *L. iners* was more prevalent in the third trimester. Regarding race and ethnicity, this is consistent with other African studies indicating that *L. iners*-dominant microbiome CST-III is most prevalent among pregnant women of African descent [15, 36, 37]. In line with our results, a racioethnic diversity study comparing the vaginal microbiome profiles of 300 pregnant and 300 non-pregnant case-matched women of African, European, and Hispanic ancestry [30] found an increased prevalence of the *L. iners-dominant* microbiome in pregnancy in all groups, with a higher prevalence of this vagitype in pregnant women of African ancestry.

GBS culture-positive pregnant carriers had a less homogenous microbial structure with a significant decrease in the *Lactobacillus* abundance level. Similar results were described by previous culture-based studies [17, 20, 21] and were subsequently confirmed using high-throughput sequencing of the vaginal microbiome [22, 25, 26]. In consistence to others [22–24, 26], *Gardnerella*, *Streptococcus* (which includes GBS), *Ureaplasma*, *Corynebacterium*, *Peptostreptococcus,* and *Staphylococcus*, as well as *Peptostreptococcus anaerobius*, were identified as significant biomarkers among GBS culture-positive pregnant women. Conversely, *Lactobacillus* was the only genus detected whose relative abundance was significantly higher in GBS culture-negative status and *L. iners* and *L. coleohominis* were discovered as significant species biomarkers. In line with our results, [39] found a greater abundance of *L. iners* in normal vaginal microbiota than in dysbiota. Interestingly, unlike other lactobacilli, *L. iners* is only present in the vagina [40, 41]. Likewise, *L. coleohominis* is a vaginal *lactobacillus* species that has been detected frequently in women with normal vaginal flora [39, 42–45]. *L. coleohominis* was previously recognized as a minor member of vaginal microbiota in women without bacterial vaginosis, consistent with our findings [46].

Notably, *L. iners* and GBS are antagonistic, persistent species that adapt well to fluctuating vaginal environmental conditions [28, 47]. Both *L. iners* and GBS produce pore-forming cytolysins that destabilize the membrane integrity of the host cell. *L. iners* produces interolysin [40, 48], while GBS produces hemolysin and CAMP factor [49, 50]. Therefore, under conditions of nutrient deprivation, cytolysins allow *L. iners* and GBS to directly extract the nutrients they require from host cells. Interestingly, interolysin is active under acidic conditions and inactive at neutral pH [48]. In contrast, the expression of the *cyl* gene cluster necessary for hemolysin release is demonstrably greater under neutral pH conditions than under acidic pH conditions [32]. This supports our conclusion that GBS-positive vaginal communities were dysbiotic and contained a lower abundance of *L. iners* than GBS-negative vaginal communities. Moreover, both *L. iners* and GBS are auxotrophic for many amino acids [40, 51], indicating that they are more dependent on exogenous sources. As a result, it is simple to hypothesize the competitive lifestyle of these bacteria because they share the same environmental resources, which ultimately manifests as either eubiosis and *Lactobacillus* dominance or dysbiosis and GBS proliferation in the vaginal econiche. Interestingly, *G. vaginalis* is also a cytolysin producer which is named vaginolysin [52] and our results showed GBS-positive communities with greater abundance of *G. vaginalis*. In line with our observation, previous reports demonstrated *G. vaginalis* as a promoter for GBS vaginal colonization [53]. This emphasizes the interplay between the members of GBS-positive communities in contrast to the obvious competition between GBS-positive microbiota and lactobacilli, the natural vaginal inhabitants.

Next generation sequencing of 16S RNA marker-gene is among the popular approaches used for profiling various bacterial communities. However, this method does not infer direct functional predictions [54–57]. The 16S RNA amplicon sequences can only provide information for taxonomic profiles but not functional profiles of sampled microbiota. Prediction tools for functional profiling of human bacterial communities have been developed based on their taxonomic composition [58]. In the current study, we hypothesized associations between inferred microbial function and GBS status using PICRUSt prediction tool [55]. This is a common computational method that uses an extended ancestral-state reconstruction algorithm to provide predictions on the potential functional composition of human microbial communities based on 16S RNA amplicon sequences and reference genome databases. PICRUSt uses the classification schemes of the KEGG Orthology (KOs) [59] and Clusters of Orthologs Groups (COGs) [60] to make predictions of gene content for all microbes in the Greengenes [61] phylogenetic tree of 16S sequences. Our results showed that GBS-positive microbial communities tend to share similar characteristics to dysbiotic vaginal communities. Our functional analysis predicted GBS-positive microbial communities would have more amino acid metabolites. In line with our findings, [14] demonstrated that vaginal dysbiosis is associated with increased levels of amino acid catabolites and polyamines and decreased levels of amino acids and dipeptides. Enriched amino acid metabolism pathways among GBS culture-positive microbial communities correspond with GBS’s role as an auxotroph for most amino acids imported from their environment [51]. Moreover, GBS culture-positive microbial communities were enriched in **t**he phosphatidylinositol signaling system. Pathogen entry into host cells requires the activation of lipid kinases, such as phosphoinositide 3-kinases (PI3K3), which are compartmentalized in lipid rafts and activated in host cells upon bacterial growth invasion. In order to invade human endometrial cells, GBS uses lipid rafts and the phosphatidylinositol signaling pathway. This is preferred by bacteria to avoid immune clearance and the intracellular degradation pathways [62]. PI3K are essential to vaginal epithelial cell invasion [63]. They have also been reported before for other vaginal pathogens such as *Gardnerella* [64] and *E. coli* [63]. This supports our results as *Gardnerella* abundance significantly increased in our GBS-positive communities. Obviously, GBS vaginal colonization is positively correlated with other microbes in this microenvironment. GBS communication and cooperation with other members of the microbiota have been suggested before [34]. Taken together, GBS-positive vaginal communities seem to provide favorable conditions for GBS vaginal colonization and persistence. On the other hand, our functional analysis revealed pyruvate metabolism and glycolysis were enriched in GBS culture-negative microbial communities. This is expected as lactic acid is a major endproduct of pyruvate metabolism and a well-defined biomarker of a healthy vaginal ecosystem [14]. This is also consistent with the prevalence of lactobacilli which drive lowering vaginal pH. Consistent to other healthy vaginal microbial communities [14, 65], our functional analysis identified pathways involved in biosynthesis of fatty acid and unsaturated fatty acids, xenobiotic biodegradation and metabolism, translation, replication, repair, and nucleotide metabolism as unique characteristics of GBS culture-negative vaginal microbial communities. Furthermore, as phospholipid constituents, fatty acids are key structural components of the membrane [66]. According to the degree of saturation, they can also exert antimicrobial properties [67, 68]. In addition, they are vital nutrients for lactic acid bacteria [68–70]. According to reports, unsaturated fatty acids are growth stimulants and stress protectors for lactobacilli [68]. Furthermore, incorporation or biosynthesis of unsaturated fatty acids has been linked to lactobacilli oxidative stress response and acid tolerance [68, 69]. Researchers discovered that fatty acid desaturation protects cells from harmful oxygen species [69, 71]. This suggests that the GBS-negative vaginal microbiome plays a protective role against invasive pathogens via hypoxic stress and the production of unsaturated fatty acids. Enhanced pathways involved in xenobiotic biodegradation and metabolism appear to provide additional protection against invading pathogens; benzoate and aminobenzoate degradation enhance their elimination from the vagina. Through the enriched KEGG pathways of translation, replication and repair, and nucleotide metabolism, GBS culture-negative microbial communities can overcome any damage or injury with DNA repair mechanisms and growth enrichment.

The current study has several limitations which should be considered in the future. First, the small sample size which may not provide an accurate depiction of diversity and composition of vaginal bacterial community or CST distribution. Second, more detailed demographics for cases and controls would have been useful when comparing vaginal bacterial communities based on GBS status. Third, we used 16S rRNA next-generation sequencing approach to investigate the vaginal microbiome. Such technique has limited taxonomic resolution and detects only part of the vaginal community. It can be less precise at the species level. Besides, it does not provide direct information on functional genes in the sample. This information is not retrievable with 16S rRNA gene sequencing but are rather predicted [54, 56, 57]. Whole genome shotgun metagenomic sequencing (WGS) would provide more precise data on taxonomic composition and functional genes of ecosystem under study. Moreover, WGS is more powerful in identifying less abundant taxa which have proven to be biologically meaningful [54, 56, 57].

## Conclusions

In this study, we demonstrated that *L. iners* is the predominant *Lactobacillus* species of the vaginal microbiome in Egypt and was previously reported as the dominant vagitype in Africa and women of African ancestry. *L. iners*-dominant microbiomes have been previously reported as transition microbiomes that predispose pregnant women to adverse pregnancy outcomes, despite being recognized as a healthy vaginal ecosystem [47]. In addition, Africa has the highest overall prevalence of GBS colonization and the highest burden of GBS-related pregnancy complications [6]. However, to date, no GBS vaccine is available, and efforts are focused on GBS serotypes [72]. Understanding how perturbations of the vaginal microbiome contribute to pregnancy complications may result in the development of alternative, targeted prevention strategies to prevent maternal GBS colonization. We hypothesized associations between inferred microbial function and GBS status that would need to be confirmed in larger cohorts. Current *Lactobacillus* probiotic candidates appear promising for preventing GBS colonization during pregnancy [34].

## Methods

### Ethics statement

All study procedures involving human subjects were reviewed and approved by the Research Ethics Committee at the Faculty of Pharmacy, Suez Canal University, Egypt (Reference number 201811RH2). The study was conducted in accordance with all applicable ethical regulations. All participants provided their consent with knowledge.

### Participants and recruitment

A cross-sectional study was conducted for a period of 3 months (December 2018 – February 2019) at the Gynecological Clinic of the Suez Canal University Hospital. During this period, 164 consecutive healthy pregnant women in their third trimester provided vaginal swab samples. All subjects underwent GBS screening via culture. The inclusion and exclusion criteria were as stated in [30, 73]. At the time of enrollment, all subjects had presumptively normal singleton gestations without known maternal comorbidities (such as type II diabetes mellitus, gestational diabetes type A2, hypertensive disorders, or chronic medical conditions) and a minimum of 24 teeth with no more than 8 missing teeth. Pregnant women with any of the following conditions were excluded from the study: a history of cancer, a compromised immune system, a history of certain chronic diseases, medication exposure within the previous six months (e.g., antibiotics, corticosteroids, cytokines, large doses of probiotics, etc.), smoking, or moderate to high alcohol intake. Participants with ruptured membranes before 37 weeks of gestation; active herpes lesions in the vulvovaginal region, abnormal discharge, bacterial vaginosis, trichomoniasis, yeast infection, gonorrhea, herpes, or warts as indicated by the physician at the time of the visit were also excluded. No vaginal samples were collected during labor delivery or at the time of discharge. Pregnant women were not subject to vaginal pH measurement and were screened for signs and symptoms of bacterial vaginosis and excluded if they were present [73]. Out of 164 participants, 43 (43/164, 26.2%) women were positive for GBS. All participants were Egyptian. The mean age of participants was 31.66 ± 7.46 (GBS-positive 30.21 ± 6.98, GBS-negative 32.22 ± 7.60). There was no significant difference for age and parity between the two groups. Information on sexual behavior were not available. Demographic data are shown in Table 4. A subset of 22 GBS culture-positive and 22 GBS culture-negative pregnant women similar for age and parity were selected for a more detailed 16S rRNA microbiome analysis from this group (Table 1). All samples included in the 16S rRNA microbiome analysis were confirmed to show more than 1000 high-quality amplicon reads as described before [26, 30, 74].

**Table 4:** Characteristics of study participants

GBS status (number)	Age (mean ± SD)	***p value***	Parity	***p value***
Culture-positive (43)	30.21 ± 6.98	0.1330	3.14 ± 1.60	0.3664
Culture-negative (121)	32.22 ± 7.60		3.42 ± 1.77	

Two-tailed unpaired t-test was calculated to evaluate the statistical differences between GBS -positive and GBS -negative participants for age and parity. *P* value < 0.05 is considered statistically significant

### Sample collection

Vaginal samples were collected from the vaginal introitus [5]. GBS detection and culturing was done in accordance with CDC recommendations [5]. As described, one additional vaginal swab was collected for 16S rRNA microbiome analysis [73].

### DNA extraction and amplicon sequencing

DNA was extracted using DNeasy PowerSoil Kit cat no. 12888-100 (Qiagen, Valencia, CA) according to the manufacturer’s instructions. Microbiota profiling was performed according to the Illumina MiSeq standard protocol of 16S rRNA gene amplicons (https://support.illumina.com/documentation.html) by amplification of the V3–V4 regions of 16S rRNA [75] with the following primers appended to the Illumina adaptor (underlined): Forward primer 5’- TCGTCGGCAGCGTCAGATGTGTATAAGAGACAGCCTACGGGNGGCWGCAG – 3′, Reverse primer 5’- GTCTCGTGGGCTCGGAGATGTGTATAAGAGACAGGACTACHVGGGTATCTAATCC – 3′. Furthermore, negative controls were analyzed to detect the presence of contaminants in swabs, extraction reagents, and during PCR mixture set up to reduce the likelihood of contamination. IGA Technology Services performed library preparation and sequencing on Illumina MiSeq Platform (Illumina, San Diego, CA) in 300 bp paired-end mode (Udine, Italy).

### Data analysis and statistics

Sequences quality was checked using FastQC (https://www.bioinformatics.babraham.ac.uk/projects/fastqc/). Quantitative Insights into Microbial Ecology (QIIME, version 1.9.1) pipeline [76] was used to prepare Illumina MiSeq sequences for 16S rRNA microbiome analysis. Poor quality sequences were trimmed using QIIME script split_liberaries.py. Using closed-reference OTU picking against the Greengenes database [61] version 13.8 with UCLUST, taxonomic assignment of Operational taxonomic units (OTUs) with 97% sequence homology was performed [77]. Further taxonomic assignment was done using SILVA database [78]. Using MicrobiomeAnalyst (https://www.microbiomeanalyst.ca), downstream processing (including rarefaction, alpha-diversity, beta-diversity, taxa abundance, core microbiome) was performed [79]. We presented the results of the database showing best taxonomic assignments. MicrobiomeAnalyst was also used to perform Linear discriminant analysis (LDA) effect size (LEfSe) for taxa biomarkers) [79, 80]. *P* values less than < 0.05 and LDA scores greater than > 2 were considered statistically significant. MicrobiomeAnalyst is a web-based platform for the comprehensive analysis of common microbiome study data outputs [79, 80]. PICRUSt (phylogenetic investigation of communities by reconstruction of unobserved states) [55] and LEfSe analysis [81] through the web-based interface (http://huttenhower.sph.harvard.edu/galaxy) were used to predict enriched microbial functional attributes most likely to explain the differences between GBS culture-positive and GBS culture-negative microbial vaginal communities based on Kyoto Encyclopedia of Genes and Genomes (KEGG). This is a database resource that integrates genomic, chemical and systemic functional information where genes from completely sequenced genomes are linked to higher-level systemic functions of the cell, the organism and the ecosystem [59]. Using 16S rRNA gene sequences, associated microbial functions of vaginal microbiome can be predicted. Briefly, the Biom file generated against Greengenes database in QIIME was used as an input file in PICRUSt. Using the PICRUSt commands “normalize_by_copy_number.py”, “predict_metagenomes.py”, and “categorize_by_function.py” functional predictions were executed to level 3 (The highest functional detail in PICRUSt). The output file from PICRUSt was further submitted to LEfSe analysis through the web-based interface (http://huttenhower.sph.harvard.edu/galaxy). The discriminative feature threshold for the logarithmic LDA score was set at > 2.

### CST assignment

Illumina 16S rRNA microbiome analysis reports provided for each sample (Analysis software version 2.6.2.3, Greengenes taxonomic database) were used for CST assignment. Vaginal bacterial communities were categorized into 5 CSTs: *L. crispatus* dominated CST-I, *L. gasseri* dominated CST-II, *L. iners* dominated CST-III, *Lactobacillus*-poor CST-IV, and *L. jensenii* dominated CST-V according to [16] based on the taxon with the largest proportions of reads. Vaginal communities dominated by a *Lactobacillus* species other than those described by [16] were categorized as CST-Other [82]. A sample was not designated a CST (No Type) when the proportion of the taxon with the largest reads is less than 30% [30, 83]. Vaginal bacterial communities characterized by the almost exclusive presence of *Gardnerella vaginalis* were assigned as CST-VI whereas CST-VII was assigned with the presence of high or approximately even proportion of *Gardnerella* and *Lactibacillus* species [84].

### Data availability

The raw 16S rRNA sequences were deposited in the NCBI database with SRA under Bio project Accession number PRJNA833599. This information is accessible at https://www.ncbi.nlm.nih.gov/bioproject/PRJNA833599.

## Supplementary Information

**Additional file 1:** **Supplementary Fig. 1.** Rarefaction curves. Samples were rarefaied to even sequencing depth. Goods coverage was above 99.9% at 97% similarity cutoff indicating that sequences richness was sufficient for vaginal communities under test in all libraries.

**Additional file 2: Supplementary Fig. 2.** Alpha-diversity indices of the vaginal microbiota across GBS culture-negative and GBS culture-positive pregnant Egyptian women using SILVA database. **A** Box plot of Shannon alpha-diversity index (*p* value < 0.01). **B** Box plot of Simpson alpha-diversity index (*p* value < 0.001). **C** Box plot of Chao1 alpha-diversity index (*p* value = 0.086554). Mann-Whitney test and Kruskal-Wallis Test were used to define statistically significant differences between GBS culture-negative and GBS culture-positive pregnant women. A *p* value < 0.05 was considered statistically significant. Astreks indicate *p* values < 0.05.

**Additional file 3: Supplementary Fig. 3.** Beta-diversity measurement of the vaginal microbiota across GBS culture-negative and GBS culture-positive pregnant Egyptian carriers using SILVA database. 2D PCoA clustering plot. Ellipses denote significant clustering Beta-diversity was assessed by PERMANOVA using Bray-Curtis dissimilarity matrix (F value = 6.1763; R squared = 0.1282; *p* value < 0.001; [NMDS] Stress = 0.15225). Each dot represents one sample.

**Additional file 4: Supplementary Fig. 4.** Species taxa level relative abundance in vagina of pregnant Egyptian women during the third trimester according to GBS status as revealed by Illumina 16S rRNA microbiome individual reports. Stacked bar charts represent relative proportions of the most predominant Lactobacillus species, Gardnerella, and non-Lactobacillus species in the vaginal microbiome of (**A**) GBS culture-negative pregnant women and (**B**) GBS culture-positive pregnant women. Each bar represents one sample.

## Data Availability

The raw 16S rRNA sequences were deposited in the NCBI database with SRA under Bio project Accession number PRJNA833599. This information is accessible at https://www.ncbi.nlm.nih.gov/bioproject/PRJNA833599.

## Abbreviations

ADI: Arginine deiminase system
CDC: Centers for Disease Control and Prevention
CST: Community state types
CydABCD: Cytochrome bd quinol oxidase system
*E. coli*: *Escherichia coli*
*G. vaginalis*: *Gardnerella vaginalis*
GBS: Group B Streptococcus
KEGG: Kyoto Encyclopedia of Genes and Genomes pathways
*L. coleohominis*: *Lactobacillus coleohominis*
*L. crispatus*: *Lactobacillus crispatus*
*L. gasseri*: *Lactobacillus gasseri*
*L. iners*: *Lactobacillus iners*
*L. jensenii*: *Lactobacillus jensenii*
*L. johnsonii*: Lactobacillus *L. johnsonii*
LDA: Linear discriminant analysis
LEfSe: Linear discriminant analysis (LDA) effect size
OTU: Operational taxonomic units
PI3K3: Phosphoinositide 3-kinases
PTS: Phosphotransferase system
QIIME: Quantitative Insights into Microbial Ecology
*S. agalactiae*: *Streptococcus agalactiae*
WGS: Whole genome shotgun metagenomic sequencing
