# Supplementary figures and images for: Alterations of the vaginal microbiome in healthy pregnant women positive for group B Streptococcus colonization during the third trimester
Source: BMC Microbiol. 2022 Dec 21;22:313. doi: 10.1186/s12866-022-02730-8 (PMC9769055; doi:10.1186/s12866-022-02730-8)

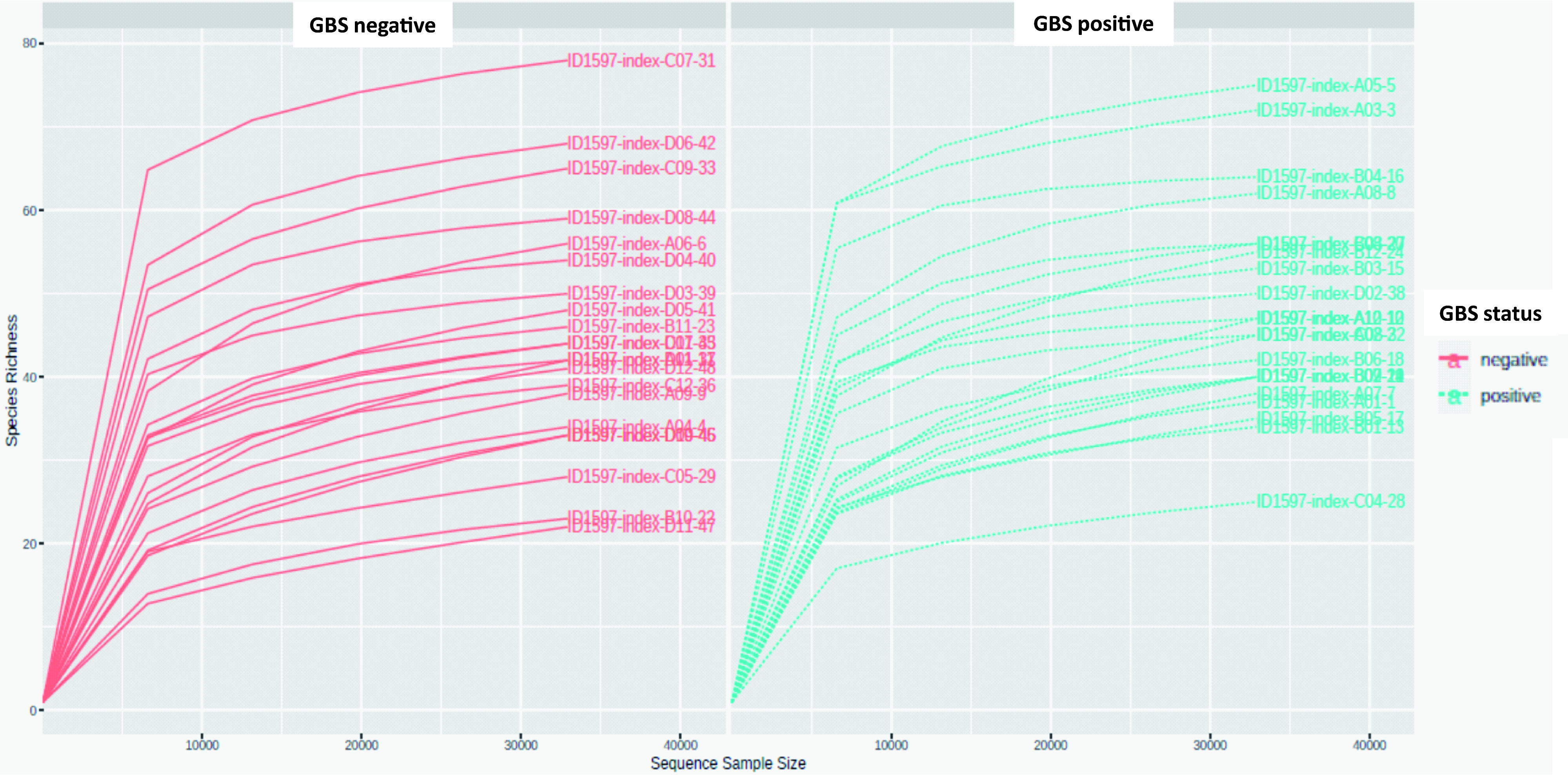

Supplement: Supplementary file 1 — Additional file 1: Supplementary Fig. 1. Rarefaction curves. Samples were rarefaied to even sequencing depth. Goods coverage was above 99.9% at 97% similarity cutoff indicating that sequences richness was sufficient for vaginal communities under test in all libraries. [file 12866_2022_2730_MOESM1_ESM.tif]

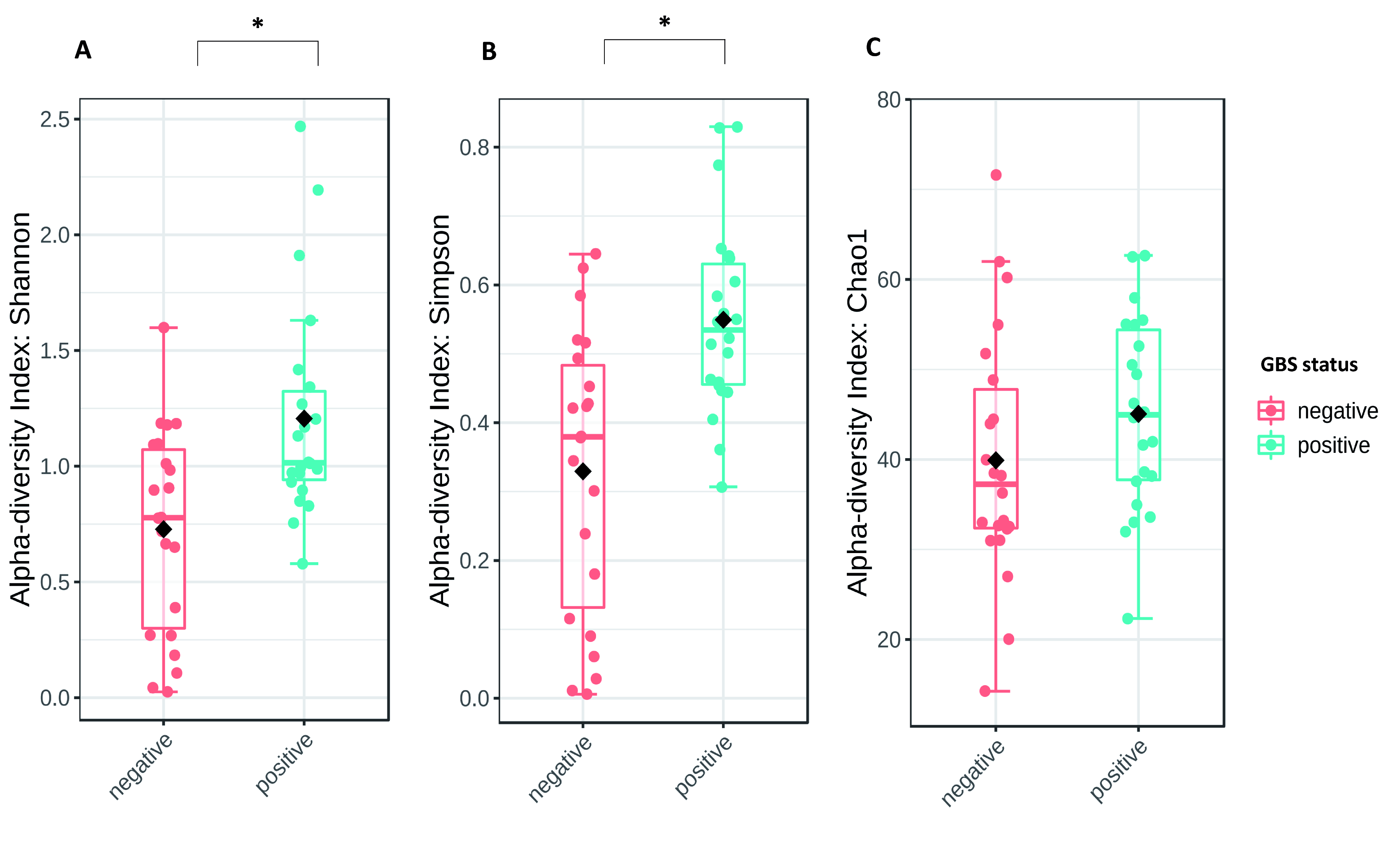

Supplement: Supplementary file 2 — Additional file 2: Supplementary Fig. 2. Alpha-diversity indices of the vaginal microbiota across GBS culture-negative and GBS culture-positive pregnant Egyptian women using SILVA database. A Box plot of Shannon alpha-diversity index (p value < 0.01). B Box plot of Simpson alpha-diversity index (p value < 0.001). C Box plot of Chao1 alpha-diversity index (p value = 0.086554). Mann-Whitney test and Kruskal-Wallis Test were used to define statistically significant differences between GBS culture-negative and GBS culture-positive pregnant women. A p value < 0.05 was considered statistically significant. Astreks indicate p values < 0.05. [file 12866_2022_2730_MOESM2_ESM.tif]

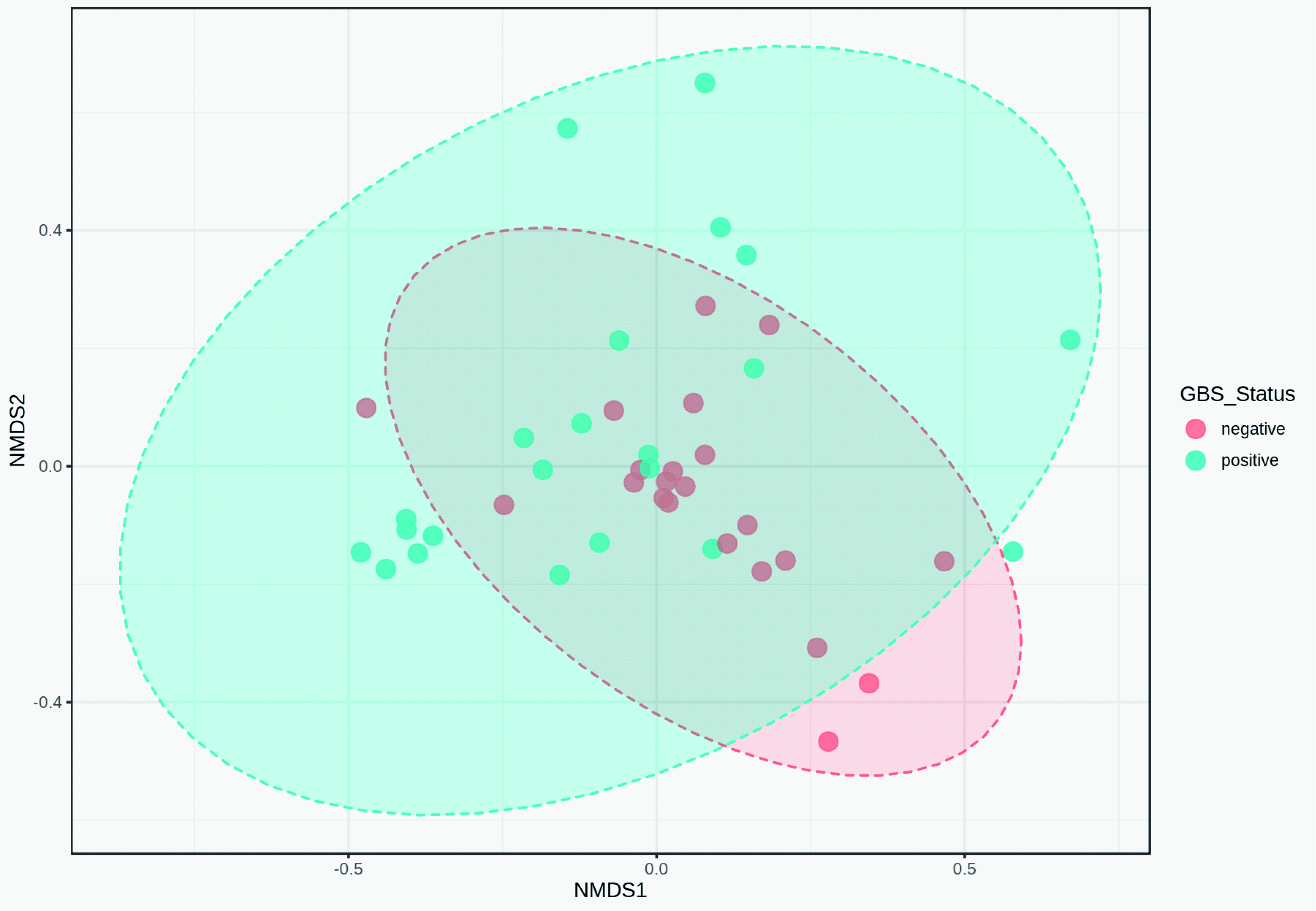

Supplement: Supplementary file 3 — Additional file 3: Supplementary Fig. 3. Beta-diversity measurement of the vaginal microbiota across GBS culture-negative and GBS culture-positive pregnant Egyptian carriers using SILVA database. 2D PCoA clustering plot. Ellipses denote significant clustering Beta-diversity was assessed by PERMANOVA using Bray-Curtis dissimilarity matrix (F value = 6.1763; R squared = 0.1282; p value < 0.001; [NMDS] Stress = 0.15225). Each dot represents one sample. [file 12866_2022_2730_MOESM3_ESM.tif]

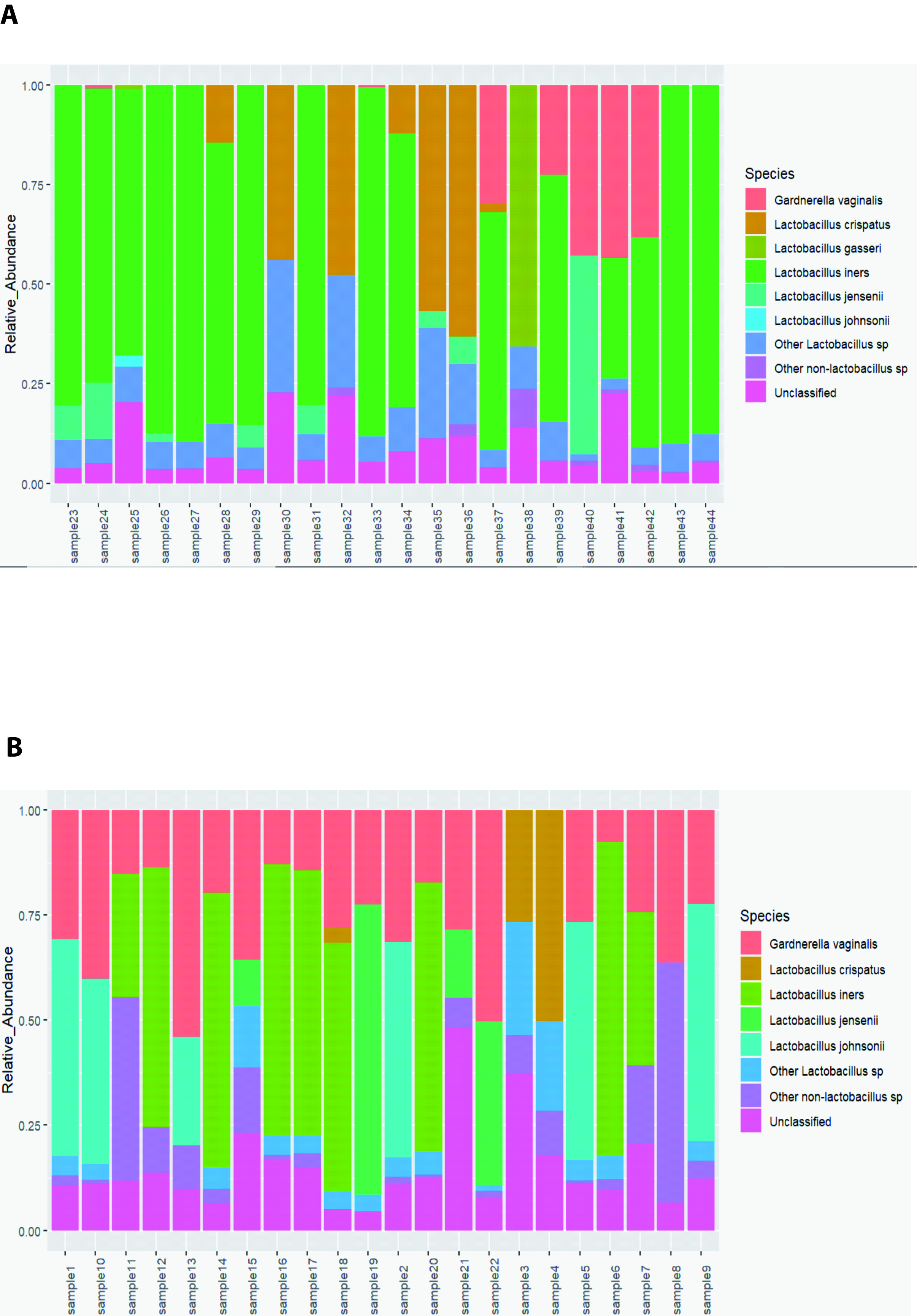

Supplement: Supplementary file 4 — Additional file 4: Supplementary Fig. 4. Species taxa level relative abundance in vagina of pregnant Egyptian women during the third trimester according to GBS status as revealed by Illumina 16S rRNA microbiome individual reports. Stacked bar charts represent relative proportions of the most predominant Lactobacillus species, Gardnerella, and non-Lactobacillus species in the vaginal microbiome of (A) GBS culture-negative pregnant women and (B) GBS culture-positive pregnant women. Each bar represents one sample. [file 12866_2022_2730_MOESM4_ESM.tif]
